# Identification of confounder in epidemiologic data contaminated by measurement error in covariates

**DOI:** 10.1186/s12874-016-0159-6

**Published:** 2016-05-18

**Authors:** Paul H. Lee, Igor Burstyn

**Affiliations:** School of Nursing, PQ433, Hong Kong Polytechnic University, Hung Hom Kowloon, Hong Kong; Department of Epidemiology and Biostatistics, Dornsife School of Public Health, Drexel University, Philadelphia, USA; Department of Environmental and Occupational Health, Dornsife School of Public Health, Drexel University, Philadelphia, USA

**Keywords:** Causal effect, Change-in-estimate, Confounding, Simulation, Model-selection, Epidemiology

## Abstract

**Background:**

Common methods for confounder identification such as directed acyclic graphs (DAGs), hypothesis testing, or a 10 % change-in-estimate (CIE) criterion for estimated associations may not be applicable due to (a) insufficient knowledge to draw a DAG and (b) when adjustment for a true confounder produces less than 10 % change in observed estimate (e.g. in presence of measurement error).

**Methods:**

We compare previously proposed simulation-based approach for confounder identification that can be tailored to each specific study and contrast it with commonly applied methods (significance criteria with cutoff levels of *p*-values of 0.05 or 0.20, and CIE criterion with a cutoff of 10 %), as well as newly proposed two-stage procedure aimed at reduction of false positives (specifically, risk factors that are not confounders). The new procedure first evaluates potential for confounding by examination of correlation of covariates and applies simulated CIE criteria only if there is evidence of correlation, while rejecting a covariate as confounder otherwise. These approaches are compared in simulations studies with binary, continuous, and survival outcomes. We illustrate the application of our proposed confounder identification strategy in examining the association of exposure to mercury in relation to depression in the presence of suspected confounding by fish intake using the National Health and Nutrition Examination Survey (NHANES) 2009–2010 data.

**Results:**

Our simulations showed that the simulation-determined cutoff was very sensitive to measurement error in exposure and potential confounder. The analysis of NHANES data demonstrated that if the noise-to-signal ratio (error variance in confounder/variance of confounder) is at or below 0.5, roughly 80 % of the simulated analyses adjusting for fish consumption would correctly result in a null association of mercury and depression, and only an extremely poorly measured confounder is not useful to adjust for in this setting.

**Conclusions:**

No *a prior* criterion developed for a specific application is guaranteed to be suitable for confounder identification in general. The customization of model-building strategies and study designs through simulations that consider the likely imperfections in the data, as well as finite-sample behavior, would constitute an important improvement on some of the currently prevailing practices in confounder identification and evaluation.

**Electronic supplementary material:**

The online version of this article (doi:10.1186/s12874-016-0159-6) contains supplementary material, which is available to authorized users.

## Background

In the practice of epidemiology, researchers identify confounders theoretically or empirically. Theoretical identification is generally carried out through use of directed acyclic graphs (DAGs) [1]. While the use of DAGs has many virtues (such as explicit declaration of hypotheses and theoretical analysis that can guide model-building in a manner that increases the possibility of empirically estimating causal association), they are subjective interpretations that reflect an investigator’s belief of how the world works, and does not necessary reflect how the world actually is [2]. As such, relying on theory alone for confounder identification is perilous: if we knew all causal relations of interest and could draw perfect DAGs, then there would be no need to empirically identify the confounders.

We focus specifically on a problem of identification of a true structural confounder present in data-generation process, i.e. a variable that would still be a cofounder if sample size was infinite, from a finite sample situation that can give rise to confounding by chance. Structural confounding is to be contrasted with confounding that arises by chance in finite samples. Such confounding by chance can be due to an association between a variable with an outcome, when such a variable is independent of exposure in population but not a sample. In such situations, it is important to be able to realize that confounding is a quirk of a finite sample, even if “controlling” for covariate in a regression model has measurable impact on exposure-outcome association. In essence, not every variable that has influence on the magnitude of exposure-outcome association in a finite sample is a structural confounder, and vice versa. It is important to correct exposure-outcome association for the peculiarities of the finite sample but one has to be cautious about generalizing that any variable identified in such a manner is a structural confounder rather than and “incidental” confounder. Distinguishing between the two types of confounding is helpful for understanding how factors under study inter-related in the population since it is the valid inferences about the population that drive application of epidemiology to policy. We attempt to address this issue in our work. However, it seems prudent to reiterate before any further analysis that it is sensible to include all know risk factors in any regression analysis of exposure-outcome association in epidemiology in order to guard against confounding by chance: application of DAG methodology can be most helpful in this regard because it allows to codify what is already known about the problem. Conceptually, any model fitted to the data has to reflect our understanding of the phenomena under study and that includes what we know already (factors forced into the model) and what we hope to learn from the data (factors that are tested the model). Thus, we always adjust risk of cancer for age and risk of autism for sex, because to do otherwise amounts to making a statement about data-generating mechanism that is known to be wrong.

Empirical confounder identification is useful when the true causal relations between the exposure, outcome, and a set of potential confounders are unknown. This is typically carried out with significance criterion, e.g., a *p*-value cutoff (≤0.05 and 0.2 are commonly used) for the association between a potential confounder and outcome, or a change-in-estimate (CIE) strategy, e.g., a ≥10 % CIE of the effect of exposure between models that do and do not adjust for the potential confounder [3, 4]. Practitioners of these approaches often cite papers by Mickey and Greenland [3] or Maldonado and Greenland [4]. However, even while these authors never advocated CIE practice for all data situations, it is not uncommon to see authors in the literature employing subjective *a priori* CIE cutoffs in the same manner as they might do with *p*-value significance testing, despite evidence that fixed CIE cutoffs result in unacceptable type I and type II error rates in many circumstances [5, 6]. Simulation-based CIE that are customized to each application and are meant to have pre-specified type I error rates were recently proposed [5]. The inevitable measurement error in covariates further complicates confounder identification in practice [7] as does latent confounding, the extreme case of miss-measured confounder. The topic of latent confounding has been addressed extensively with excellent proposal for analytical treatment, e.g. see [8, 9] for review.

Accurate knowledge of measurement error magnitude and structure is sometimes lacking in epidemiology. However, in large-scale and well-conducted epidemiological studies, researchers have to make use of measurements with known error (obtained in validity and reliability studies) to achieve the required sample size and to reduce participant burden, for example self-report of dietary intake instead of a blood test [10–13]. The effects of measurement errors in exposures and confounder on the performance of different confounder identification criteria are unknown, although insights exist on bias and variability of estimates in such cases, albeit with closed form solutions currently for linear regression only [14]. When measurement error is not known exactly, researchers may still conduct sensitivity analysis to see how choice of confounder identification strategy may bias the results; we illustrate this in the applied example in this paper. There may be a range of plausible measurement errors magnitudes that has negligible influence on confounder identification strategy. It is also important to know that epidemiologists always have some intuition about the accuracy of their tools and are aware that most are imperfect, otherwise they would not be able plan their work at all.

The primary aim of removing cofounding from the estimate of exposure of interest on the outcome is to obtain unbiased estimate of the degree of exposure-outcome association that can be useful in risk assessment. This is indeed the conceptual foundation of CIE approach that proposed cut-offs on the order of 10 % as these were judged to be reflective of what can be reliably inferred in observational epidemiology given limitations of the data. From this perspective, it also acceptable to force all potential covariates into disease model so long as they are suspected as *potential confounders* and can be ruled out as factors that should not be adjusted for (e.g. mediators, antecedents of exposure alone, etc.) on the basis of theoretical analysis (e.g. implemented via DAG). This is so because if regression-based adjustment has negligible effect on estimate of interest, there is equally no harm in the adjustment so long as the model is not over-fitted. However, there is also virtue in understanding whether there is evidence that a specific factor is a confounder, e.g. in cases where such a factor is “costly” to assess and one is planning future work on a particular topic and wishes to optimize study protocol. In recognition of importance of accurate estimate of causal effects in epidemiology, rather than hypothesis testing, we also consider influence of different confounder-selection strategies on accuracy of the estimate of the exposure-outcome association.

Here, we illustrate a mixed approach for confounder identification utilizing both theoretical and empirical criteria that accounts for the realistic role of measurement error in the exposure and putative confounder, along the lines suggested by Marshall [15]. While using both theoretical and empirical criteria for model selection has been proposed [16], we provide a simulation-based framework that evaluates the performance of various empirical criteria. We also address the issue of confounding by a risk factor by chance in finite sample by proposing a modification on the previously proposed simulation-based CIE approach. Next, we demonstrate the application of CIE criteria in a real-world study of mercury and depressive symptoms, and where theory can be injected into the process to optimize causal inference.

## Methods

### Empirical confounder identification strategies

#### Overview

Five strategies were used, namely significance criteria with cutoff levels of *p*-values fixed at ≤0.05 and 0.2 (in which a putative confounder is adjusted for if the *p*-value of the *t*-test of the null hypothesis testing its effect on outcome equals zero is smaller than the cutoff levels), and CIE criterion with three different cutoff levels (fixed *a prior* at 10 %, with type I error controlled to a desired level, and with type II error controlled to a desired level). The observed change in estimate due to covariate *Z* is calculated as *Δ*=|(*θ*_*0*_*– θ*_*Z*_)/(*θ*_*0*_)*|*, where *θ*_*0*_ is the effect estimate of interest not adjusted for suspected confounder *Z* and *θ*_*Z*_ is the effect estimate adjusted for suspected confounder *Z*. When CIE approach is used, a covariate *Z* is included in the final model if its inclusion in regression model produces *Δ* ≥ *δ*_*c*_*,* where *δ*_*c*_ is 0.1 in the 10 % CIE approach, or *δ*_*c*_ is determined by simulations as described below. We will describe simulation-based CIE approaches in more detail below, as well as pre-screening aimed to reduce confounding by a risk factor by chance.

#### Simulation-based change in estimate (CIE) approach

As a way of improving on an empirical approach with criteria fixed *a prior*, we previously proposed a simulation-informed CIE strategy [5] that performs better in confounder identification and causal effect estimation [17]. In brief, the simulation-informed CIE criterion determines change in the effect estimate of interest that arises when the exposure of interest is adjusted for an independent random variable. With this approach, an independent random variable with the distribution identical to the observed putative confounder is drawn and the causal effect estimates of the exposure and outcome adjusting and not adjusting for this independent random variable are obtained. Next, we record the change-in-estimate that results from adjusting this independent random variable. The above procedure is repeated and the resulting distribution of changes in effect estimates upon adjustment indicates where we need to place a cut-off for the CIE criterion in order to achieve the desired type I error, e.g. for 5 % error the 95 %-percentile of the distribution is used. One can also adopt a CIE criterion with a desired type II error. To do so, one repeatedly simulates a variable with particular correlations with the exposure and outcome, and compares the CIE from models that do and do not adjust for this simulated confounding variable. Using the *s*th-percentile of the simulated CIEs as a cutoff could yield a type II error of 1-*s*. In our simulations, we focus on selection of these two CIE cutoffs. In the next section, we describe this procedure in more detail, infusing it with consideration of measurement error.

#### Screening potential structural confounders

In preliminary investigations, application of simulated CIE approach resulted in an unacceptably high rate (e.g. 50–80 % in some instances) of identification of a risk factor as a structural confounder when it was in fact not correlated with exposure of interest in the population (i.e. by data generating process). We identified correlation of exposure and covariate in finite samples as the culprit of this artifact and developed a screening step that evaluated correlation of exposure and putative confounder before evaluating it further via the five strategies described above. Specifically, only if the hypothesis that the observed exposure and covariate were not correlated was rejected, then the covariate was considered further in the identification of structural confounding. On the other hand, if the hypothesis that the observed exposure and covariate were not correlated was not rejected, then the covariate was excluded from further evaluation in the identification of structural confounding.

### Simulation study: overall framework and method of analysis

In specific simulations that we undertook, we assumed that (a) the exposure is associated with the outcome and (b) the putative confounder is indeed a confounder by virtue of its association with both exposure and the outcome (but not the descendant of them). As in many real-life situations, the exposure and confounder are measured with error: for simplicity, we focus on additive classical measurement error models with uncorrelated errors (but our simulation framework can readily be amended to accommodate more complex situations).

The disease model that was considered in our investigation was of the form *g*(*Y)* = α + *βX + γZ*, with *g()* representing the link function of the generalized linear model, the fixed effects α (background rate or intercept), *β* (influence of exposure *X* on outcome *Y*)*,* and *γ* (influence of covariate *Z* on outcome *Y*). The regression coefficient *β* is only identical to true value of the effects of interest in linear regression but for logistic and Cox proportional hazard regression, the effects of interest is calculate as relative risk (RR) and hazard ratio (HR), respectively. We denote these true effects of interest as *θ* for generality.

We assumed that we can only observe realizations of true exposure and confounder with classical additive measurement error models X^*^ = *X* + *ε*_*x*_ and Z^*^ = *Z* + *ε*_*z*_, the error terms are unbiased and independent of each other. The estimates of regression coefficients *β* from (*Y*, X^*^, Z^*^) data with and without adjustment for Z^*^ are denoted by *β*_Z_^*^ and *β*_0_^*^, respectively. These regression coefficients can be used to calculate estimates of the effect of interest *θ* as *θ*_Z_^*^ and *θ*_0_^*^, with and without adjustment, respectively; the superscript “^*^” denotes variables and estimates contaminated by measurement error.

The screening test for Pearson correlation of X^*^ and Z^*^ being different from zero used *p*≤0.05 cutoff. The datasets where covariates *Z* were not rejected are evaluated using the simulated CIE cutoff calculated as follows.

The simulated CIE cut-offs in presence of measurement error are determined by comparing effect estimates relating X^*^ to *Y* with and without adjusting regressions of *Y* on X^*^ for an independent random variable *Z*_*0*_ with distribution identical to that of Z^*^ over *K* simulations. Let us denote such effect estimates, functions of regression coefficient, as *θ*_0_^*^_k_ when unadjusted coefficient is used, and as *θ*_Z0_^*^_k_ when the adjusted coefficient for *Z*_*0*_ (not the same as adjusted for *Z*) is used in the *k*^*th*^ simulation. Then, the changes in the estimates in each simulation are then calculated, in general, as

$$ {\delta}_k = \Big|\left({\theta}_0{{}^{*}}_k\hbox{--}\ {\theta}_{Z0}{{}^{*}}_k\right)/\left({\theta}_0{{}^{*}}_k\right)\Big| $$

and the *q*^th^-percentile of *δ*_*k*_ determined over *K* simulations the cutoff for CIE that would lead to a type I error of 1-*q*, i.e. *δ*_*c*_*.* The CIE that is simulated to achieve desired type II error can be obtained in a similar manner, with the independent random variable *Z*_*0*_ replaced by a random variable correlated with X^*^ and Z^*^ according to the simulation setting (i.e. under the assumption that we guessed correctly the true nature of associations in data-generating process), and the *s*^th^ percentile of *δ* determined over *K* simulations the cutoff for CIE that would lead to a type II error of *s*. As with all power calculations, this requires an informed guess of the structure we aim to detect and is therefore the more difficult criteria to establish objectively (e.g. we do not know true value of all the correlations from the data contaminated by measurement error) as opposed to the one that strives to control type I error.

### Simulation study: the specific scenarios

Our example synthetic data scenario features an outcome *Y* and three different types of outcome were generated, namely binary (with the disease prevalence at follow-up of 10 %), continuous (with a variance of 1), and survival (with the death rate at follow-up of 10 %). The exposure *X* and true confounder *Z* both simulated to follow standard normal distributions, *Z* is associated with both *X* (via Pearson correlation *ρ* ≠ 0) and *Y* (*γ* ≠ 0). The binary, continuous, and survival data were generated and fitted using a logistic model (ln(*P*(*Y* = 1)/*P*(*Y* = 0) = ln(1/9) + *βX + γZ*)), a linear model (*Y* = *βX + γZ + ε*_*y*_, *ε*_*y*_ *~ N*(0, 1)), and a Cox model (survival time ~ exp(*βX + γZ*-min(*βX + γZ*)), censored at survival time > 0.1), respectively. The survival times were generated as follows: (1) mean survival time for all subjects equaled *βX + γZ*, (2) the aforementioned means survival times were linear transformed to make then all positive by subtracting the minimum value of (*βX + γZ*), (3) the survival time for each subject was generated to follow an exponential distribution with rate parameter equal to mean survival time from step (2), (4) survival times were censored at a value of 0.1 so that the outcome was observed in only 10 % of subjects.

In illustrating the kind of information that this tool can yield, we obtained *N* = 10,000 simulation realizations of a cohort study (yielding a standard error of 0.5 %) of either *n* = 500 or 2,000 subjects, with *ρ*∈{0.1, 0.2, 0.3, 0.4, 0.5, 0.6, 0.7, 0.8, 0.9} and the true causal associations of *X-Y* and *Z-Y* with *β = γ* =0.1, as well as a situation in which exposure of interest is measured with smaller error than or equal to the putative confounder, i.e. *ε*_*x*_ *~ N*(0, σ_x_^2^∈{0.25, 1}) and *ε*_*z*_ *~ N*(0, σ_z_^2^∈{0.25, 1}). We used *K* = 10,000 to determine simulation-based CIE for each combination of parameters defined a simulation framework above.

In each *n*^*th*^ simulation realization (1, …, *N*), when screening potential confounders, we evaluated Pearson correlation of X^*^ and Z^*^ (*ρ*_n_^*^) and rejected Z^*^ as potential confounder when *p*-value of the null hypothesis *ρ*_n_^*^ *=* 0 was larger than 0.05. In such instances, final model selected excluded Z^*^. If Z^*^ remained in contention for role of structural confounder after the screening text, we next estimate the effects of X^*^ on *Y* in simulated datasets by

- (a) fitting a regression model appropriate for each outcome with X^*^ as an independent variable and *Y* as the dependent variable (i.e., do not adjust for Z^*^), resulting in estimate of effect of *X* on *Y* as *θ*_0_^*^_n_, which is a function of *β*_0_^*^_n_, and
- (b) fitting a regression model appropriate for each outcome with X^*^ and Z^*^ as independent variables and *Y* as the dependent variable (i.e., adjust for Z^*^), resulting in estimate of effect of X^*^ on *Y* as *θ*_Z_^*^_n_, which is a function of *β*_Z_^*^_n_*.*

Effect estimate and *p*-values resulting from these models of the *n*^*th*^ simulation realization are compared and, depending on the confounder selection rule that was triggered, the final estimate of the effect of *X* on *Y* in that particular simulated dataset was computed by either model (a) or (b).

We also calculated root mean squared error (RMSE) of effect

$$ \sqrt{{\displaystyle \sum_{n=1}^N{\left({\varphi}_n-\theta \right)}^2/N}} $$,

where in the *n*^th^ simulation (*n* ∈ {1,…,*N*}) *φ*_*n*_ = *θ*_Z_*_n_ if the confounder identification criteria suggested an adjustment, and *φ*_*n*_ = *θ*_O_*_n_ otherwise; recall that *θ* is the true value of the effect estimate set by simulation.

All simulations were carried out using R 3.2.0. The R code we provide allows one to test various CIE cutoffs in order to determine the percentage of the simulated datasets correctly identifying Z^*^ as a confounder or effect of X^*^ as significant, as well as RMSE resulting from model selected after application of each confounder-identification strategy (Additional file 1).

### Application to study design to clarify role of a confounder in NHANES

We illustrate the application of our approach in an example arising from an earlier analysis of exposure to total blood mercury (*E*) and depression (*D*) in 6,911 adults aged ≥40 years in the National Health and Nutrition Examination Survey (NHANES) 2009–2010 [18] approved by The National Center for Health Statistics Research Ethics Review Board; informed consent was obtained from all participants at the time of data collection and further consent for specific analyses of this publically available data is not needed. The dataset can be downloaded at http://wwwn.cdc.gov/Nchs/Nhanes/Search/nhanes09_10.aspx.

**Fig. 1:**
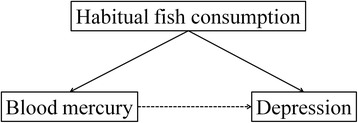
Direct acyclic graph of the causal effect between blood mercury and depression

Contrary to an expected null association between exposure and outcome, a nonsensical inverse (protective) association was reported and the original investigators argued that this was probably due to measurement error leading to residual confounding by fish consumption (*F*) [18]. That study assessed the binary recall of fish consumption in the 30 days prior to data collection (*F*_*obs*_). This variable does not demonstrate statistical properties that support its inclusion as a confounder in the final model because (a) *p*-value for *F*_*obs*_ in the logistic regression model = 0.82, and (b) inclusion of *F*_*obs*_ the final models does not affect the observed RR_*ED* | *Fobs*_ = 0.83 (OR_*ED* | *Fobs*_ = 0.79) of depression due to mercury to the third decimal place. Nonetheless, it is important to note that our preliminary test for potential confounding would not have rejected *F*_*obs*_ from the final model because there is evidence that it is correlated with exposure to mercury, albeit “weakly”: Pearson correlations of 0.39 with mercury exposure (95 % CI 0.37–0.41, *p*< 0.05). Furthermore, given the established effects of habitual fish consumption *F* on blood mercury *E* [19] and depression *D* [20], Ng et al. [18] suspected that *F* is a confounder of association of total blood mercury with depression (see Fig. 1 for the DAG of the causal association), and that the pattern of results arose because *F*_*obs*_ is a poor measure of unobserved *F*.

Let us suppose that in the course of future research we have a particular method of measuring fish consumption (*W*) with a known degree of measurement error that may prove to be superior to *F*_*obs*_. It is important to note that we do not wish to use *F*_*obs*_ in the new research project with the same sample: it yielded what we believe to be confounded estimates and the motivation of new research would be to improve on quality of data to understand the problem better. We need to simulate *W* because it is not given in the data but is a reflection of what we can hope to obtain in the study that is being planned under the assumption of given causal structure: we can never hope to measure *F* itself but need to be generate *W* and thereby evaluate performance of *W*. We want to know two things: (a) whether *W* is a confounder of the mercury-depression relationship, and (b) whether models adjusted for this measure of fish consumption *W* will result in the hypothesized null mercury-depression association (i.e., RR_*ED* | *W*_ = 1, as opposed to the observed estimate RR_*ED* | *Fobs*_ = 0.83) Here, *W* is related to true habitual fish consumption *F* by classical measurement error: *W* = *F* + *ε*, *ε* ~ *N*(0, *σ*^*2*^); *F ~ N*(0,1); *ε* is independent of both *E* and *F*. To more specifically motivate these assumptions, reflecting on common experience in exposure assessment, we consider that *F* is a measure of fatty acid intake that is measured by a biomarker and then normalized to Gaussian distribution via log-transformation, hence additive measurement error model for *W* and distributional assumptions can be appropriate. In practice, such assumptions would be verified in a validation or reliability study.

We assumed that total blood mercury *E* is near-perfectly measured because a precise analytical technique exists. To simplify, we ignored the matter of etiologically relevant windows of exposure, although this may not be trivial because the biologic half-life of mercury in blood is short.

Based on prior research [18], we also assumed: (a) the association between *F* and *D* is based on the correlation of underlying continuous measures of *F* and *D*, and set it to *ρ*_*FD*_ *= −*0.35, and (b) that the correlation of *F* and *E* is *ρ*_*FE*_ *=* 0.39, same as the observed correlation *F*_*obs*_ and *E*. With these inputs, we simulated true (*F*) and mis-measured (*W*) values of fish consumption subjected to different magnitudes of measurement error. Under various conditions of measurement error, we simulate *W* 10,000 times. Different degrees of error in measured confounder, *σ*^*2*^, were examined. We acknowledge that a different model for confounding could have been postulated and included in our simulation framework but we followed the path deemed as sensible by the original investigators in [18].

To empirically determine whether *measured* fish consumption *(W)* would correctly remove confounding from effect of mercury on depression, the proportions of simulated datasets in which the adjusted association of mercury on depression, RR_ED | W_, has *p* >0.05 were recorded. This is akin to asking how well should we measure confounder in order to have the power to observe true exposure-response association upon adjustment. We also reported the simulation-determined confounder identification criterion described above (i.e. aimed to control type I error at 5 %) to compare it to the conventional CIE of 10 %. Finally, we also determined the average and the 95 % empirical confidence intervals of the estimates of the mercury-depression association with adjustment for simulated values of *W* based on the 10,000 simulations, in order to determine how well the adjustment for *W* is able to estimate a specified true causal effect of *E-D* adjusted for *F.* (The number of simulations we informed by the observations that it was sufficient to obtain stable behavior of simulation realizations; in every specific situation, a difference size of simulation may be needed.) This reflects a theoretical perspective for confounder identification where based on some pre-determined DAG, *W* is theoretically a confounder of the exposure-outcome association and should therefore be included in models regardless of measurement properties. To visualize the empirical distribution of RR_*ED* | *W*_, we plotted its histogram from the 10,000 simulated estimates with *σ*^*2*^ = 1. The R code for the simulations can be found in the Online Supplementary Materials (Additional file 2).

## Results

**Fig. 2:**
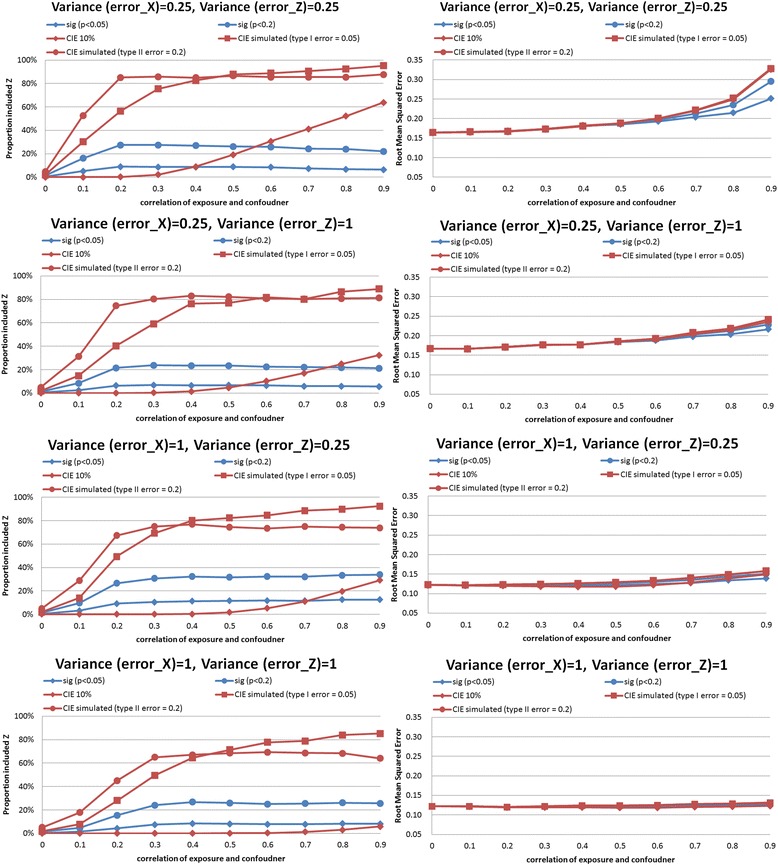
Proportion of analyses that correctly identify a confounder and root mean squared error under different empirical confounder identification strategies in a cohort study (500 subjects with binary outcome)

**Fig. 3:**
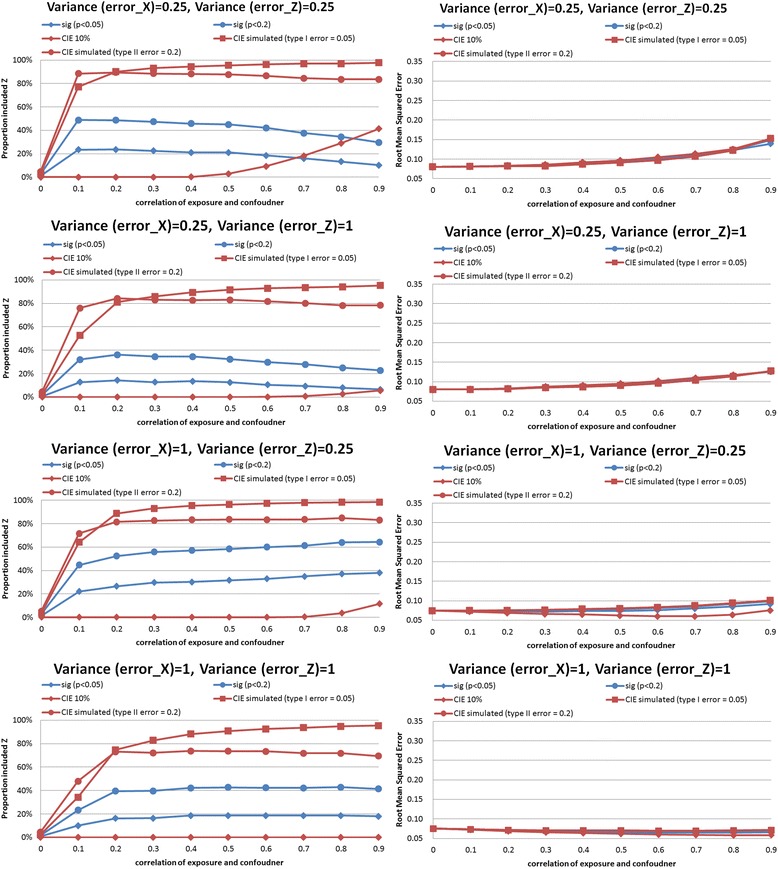
Proportion of analyses that correctly identify a confounder and root mean squared error under different empirical confounder identification strategies in a cohort study (2000 subjects with binary outcome)

**Fig. 4:**
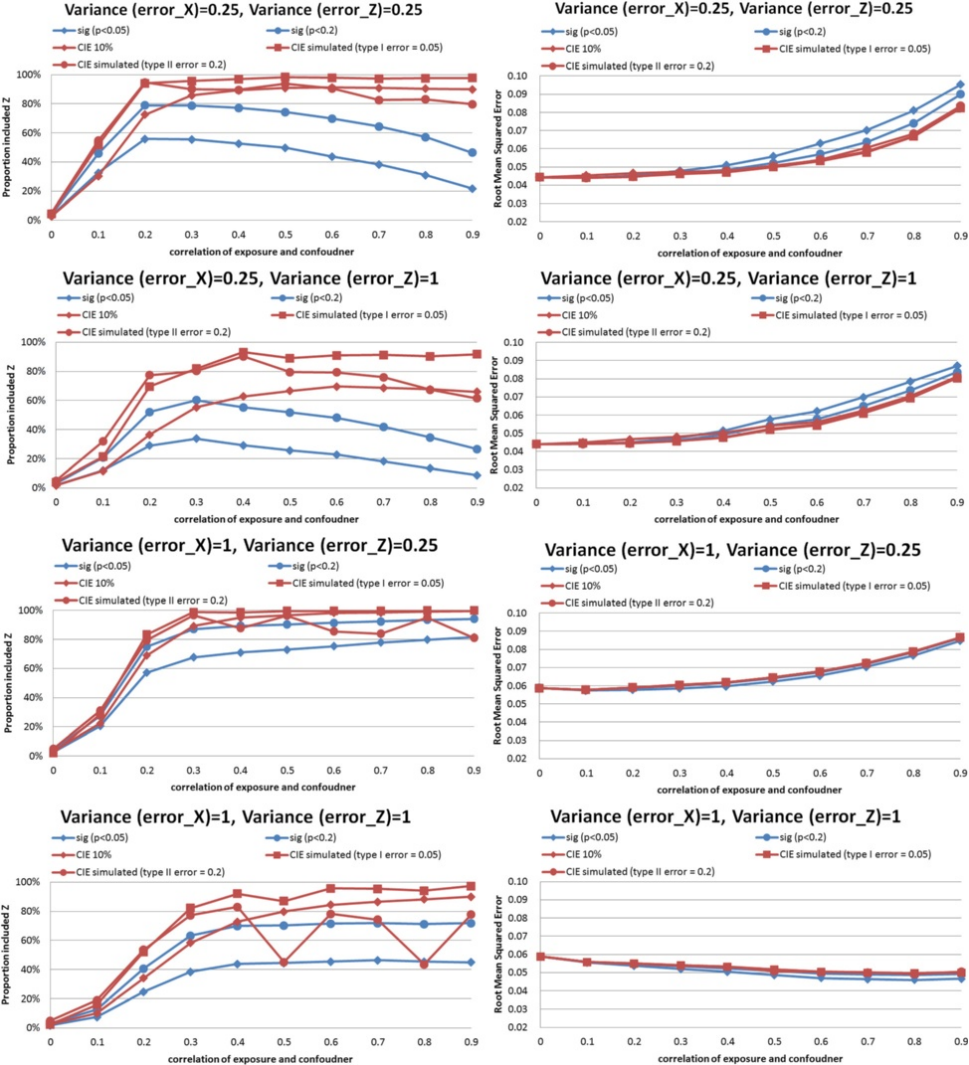
Proportion of analyses that correctly identify a confounder and root mean squared error under different empirical confounder identification strategies in a cohort study (500 subjects with continuous outcome)

**Fig. 5:**
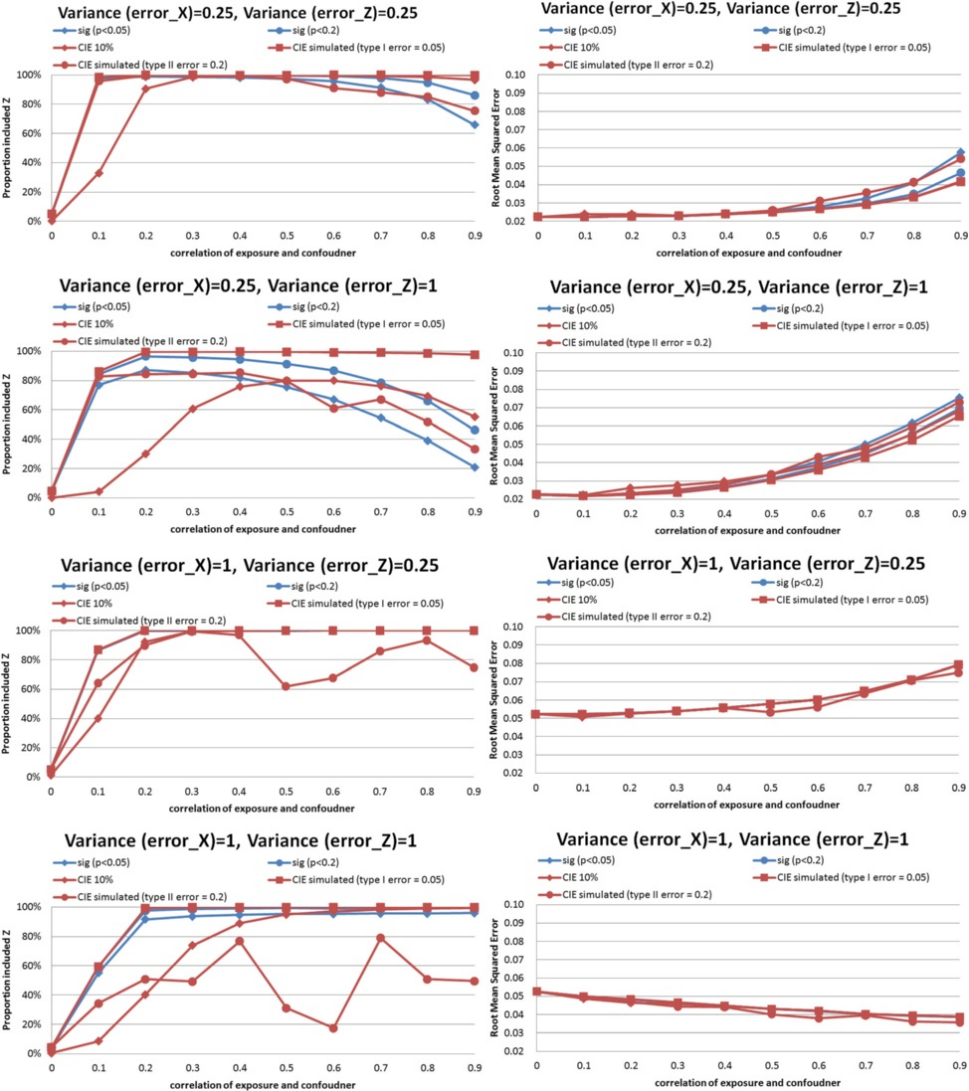
Proportion of analyses that correctly identify a confounder and root mean squared error under different empirical confounder identification strategies in a cohort study (2000 subjects with continuous outcome)

**Fig. 6:**
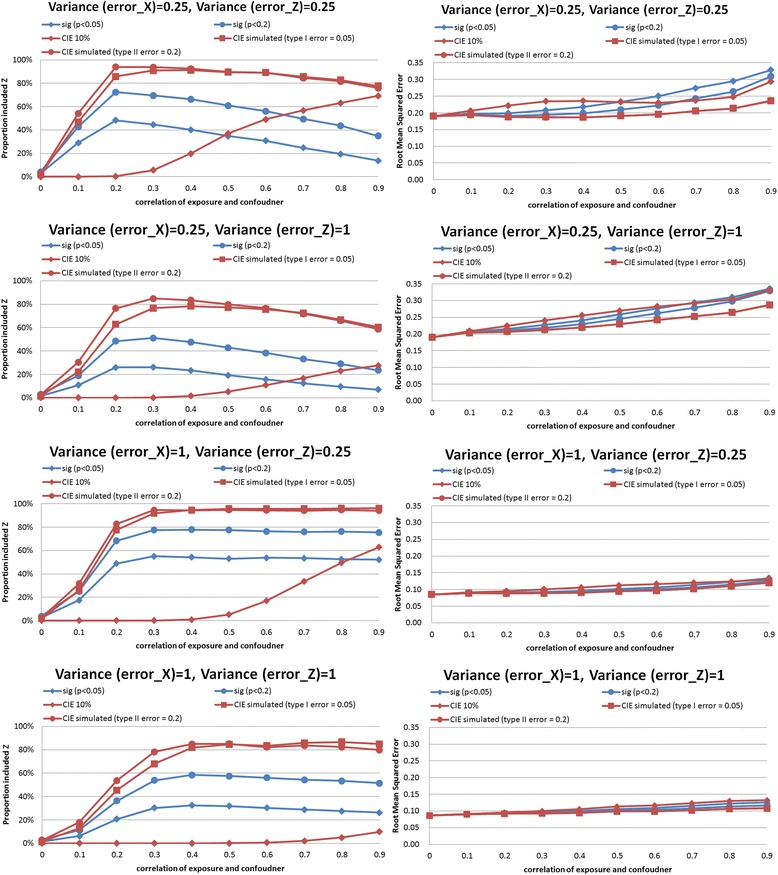
Proportion of analyses that correctly identify a confounder and root mean squared error under different empirical confounder identification strategies in a cohort study (500 subjects with survival outcome)

**Fig. 7:**
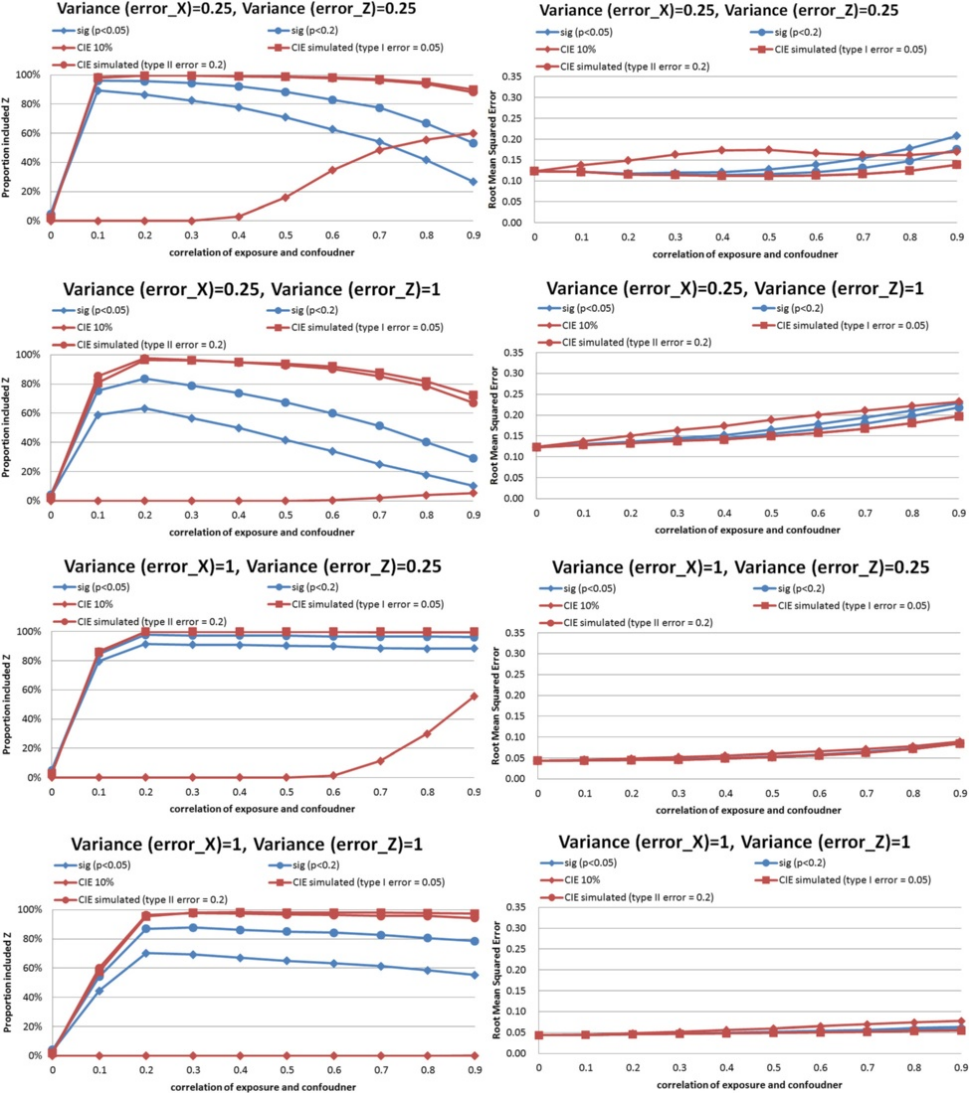
Proportion of analyses that correctly identify a confounder and root mean squared error under different empirical confounder identification strategies in a cohort study (2000 subjects with survival outcome)

In the synthetic example, we performed simulations comparing CIE between models that did and did not adjust for the confounder. Results of the simulations are shown in Figs. 2, 3, 4, 5, 6 and 7. The simulations indicated that a change in the estimate of the exposure-outcome relationship of 0.2 % (e.g. Cox model, *n* = 2,000, σ_x_^2^ = σ_z_^2^ = 1, *ρ* = 0.4) to 7.3 % (linear model, *n* =500, σ_x_^2^ = σ_z_^2^ = 1, *ρ* = 0.5) between models that do and do not adjust for the confounder is expected to result in type I error 5 % in the studied settings. The control of type II error to 20 % was achieved with simulated CIE on the order of 0.25 % (binary model, *n* =2,000, σ_x_^2^ = 0.25, σ_z_^2^ = 1, *ρ* = 0.2) to 64 % (linear model, *n* =2,000, σ_x_^2^ = 1, σ_z_^2^ = 0.25, *ρ* = 0.9). Upon further investigations, we found that the simulation-determined cutoff was very sensitive to measurement error in exposure and potential confounder; there was some tendency for an inverse association between the cutoffs but the clear pattern was only apparent for large error variances (details available upon request). For example, under the scenario of linear regression, *n* =500, *ρ* = 0.5, and σ_z_^2^ = 1, the simulation-determined cutoff with expected type I error of 5 % equaled 3.6 % when σ_x_^2^ = 0.25 and increased to 7.3 % when σ_x_^2^ = 1. We also verified that evaluation of *p*(ρ^*^ = 0) with criteria of 0.05 was important in this setting for controlling false positives. In absence of such a screening test, the rate of *Z* falsely identified as structural (rather than chance) confounder was commonly on the order of 50–80 % as seen by acceptance of Z^*^ as confounder when in fact *ρ =* 0 by simulation (details available upon request).

### Comparison of confounder identification strategies in a synthetic data: identification of structural confounder

Compared with the empirical criteria tested that were fixed *a priori* (significance criteria with cutoff levels of *p*-values of 0.05 or 0.20, and CIE criterion with a cutoff of 10 %), the two simulation-determined CIE criterion exhibited superior performance in selecting the correct model within at least 80 % of simulated datasets for exposure-confounder correlation of 0.2 or higher. In contrast, the three traditional methods perform poorly in all three outcome models.

The traditional methods identified a true confounder generally in less than 60 % of the simulated datasets with *binary outcomes* (logistic regression), even when the cohort size increased from 500 to 2,000 (Figs. 2 and 3, left-hand panels). The gap in performance in cofounder identification was more apparent for smaller cohort (Fig. 2, left-hand panels): 20–40 % drops in power for the stronger simulated confounding with *ρ*>0.5. Similar gap in performance remained when cohort was increased to 2,000 while measurement error in exposure was fixed at the lower value, and when measurement error in cofounder was greater than that in exposure (Fig. 3, top 2 left-hand panels). However, as both cohort size and measurement error in exposure increased and confounding became stronger (*ρ*>0.3), a more regular pattern of power was observed: CIE criteria simulated to control type I error had power 70–100 %, CIE criteria simulated to control type II error had power 70–80 %, significance test *p*<0.20 had power 40–60 %, significance test *p*<0.05 had power 20–30 %, and 10 % CIE criteria failed to identify structural confounder in almost all instances (Fig. 3, bottom 2 left-hand panels).

In *linear regression*, the traditional methods were more comparable to simulated-based CIE approaches but their performance depended on strength of confounding and degree of measurement error in a complex fashion. When higher value of error variance in exposure *X* were examined, all approaches had similar performance in confounder identification for the large cohort of 2000 (Fig. 6, bottom 2 left-hand panels), except that CIE method designed to control type II error to 20 % behaved erratically as strength of confounding increased beyond *ρ* = 0.3. In smaller cohort size with the same “large” error in exposure (Fig. 5, bottom 2 left-hand panels), however, there was a clear advantage to simulation-based CIE method catered to control type I error to 5 %, especially when cofounding grew stronger: it maintained power of at least 80 % beyond *ρ* = 0.3 whereas significance testing and CIE cutoff for control of type II error to 20 % were less successful, with power dropping below 80 % as both the strength of confounding (*ρ* >0.3) and measurement error in confounder increased (Fig. 5, bottom left-hand panel). The divergence in performance of different criteria was the greatest when error in confounder exceeded error in exposure and the cohort size was smaller (e.g. compare Fig. 5, 2^nd^ from top left-hand panel vs. Fig. 5, 2^nd^ from top left-hand panel).

*Survival analysis* mimicked linear model but deficiency of performance of traditional approaches tended to be greater and, paradoxically, worse with smaller measurement errors for exposure. For example, in survival analysis with cohort size of 2,000 and error variances 0.25 (the smallest tested) and strongest confounding (*ρ* = 0.9), when simulated CIE criteria correctly included *Z* as confounder in >80 % of cases, the “significance-testing” approaches had power of 30–60 % only (Fig. 7, top left hand panel). The gap in performance reduced when error variances increased to the largest value tested: 90 % vs. 60–80 % (Fig. 7, bottom left hand panel); it must be noted that the reverse pattern held for the 10 % CIE approach as its power dropped to zero as measurement error increased. It can be observed that when error in confounder increased for this setting, but error in exposure was help constant, the significance criteria suffered greatest loss of performance (from power of 90 % to <40 %) and 10%CIE criterion dropped power from about 60 % to <10 % (Fig. 7, two middle left hand panels). The patterns in smaller cohort of 500 were similar (Fig. 6).

### Comparison of confounder identification strategies in a synthetic data: precision

The simulation-determined CIE criterion achieved the smallest RMSE in all survival analyses (Figs. 6 and 7, right hand panels). In linear models, the pattern was complex. For the smaller cohort size, simulation-based CIE approaches led to smaller RMSE only when error in exposure measurement was at the lower tested value (Fig. 4, two upper right hand panels), otherwise, the significance testing approached yielded smaller RMSE (Fig. 4, two bottom right hand panels). For a larger cohort, linear model built using simulation based CIE tended to be associated with lower RMSE (Fig. 5, right hand panels). In logistic regression, simulation-based CIE approaches also tended to produce larger RMSE for the smaller of the tested cohort (Fig. 2, right hand panels), with the 10 % CIE criteria leading to the lowest RMSE across varying degrees of confounding (Fig. 2, bottom right hand panel). When a large cohort was considered in logistic regression analysis, the simulation-based approaches had lower RMSE when measurement error in exposure was fixed at a smaller value only (Fig. 3, right hand panels), just like in the linear model.

Larger degree of measurement error tended to produce lower RMSE values (e.g. survival analysis, Figs. 6 and 7, right hand panels), possibly indicating clustering of estimates around attenuated effect estimate and conveying false certainty in the effect estimate under measurement error. There was also a tendency for RMSE to increase with the degree of cofounding in most studied settings (Figs. 2, 3, 6 and 7, right hand panels) However, the RMSE deceased with the exposure-confounder correlation in linear models, when the measurement errors of exposure and confounder were both “large” (i.e. set at 1) (Figs. 4 and 5, bottom right hand panels). On the other hand, when measurement errors are smaller, in linear model there is an increase in RMSE with the strength of confounding (Figs. 4 and 5, top 3 right hand panels) as in other models.

### Influence on power of excluding a true confounder by the screening procedure

**Fig. 8:**
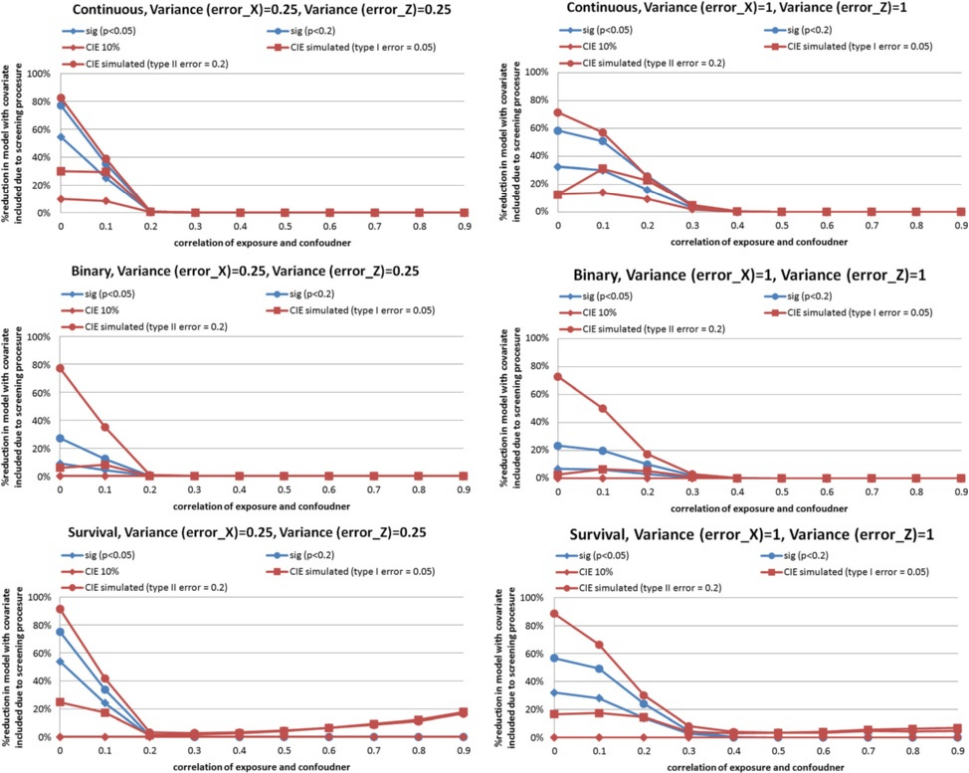
The change in the proportion of analyses that excluded a confounder due to the proposed screening procedure under different empirical confounder identification strategies in a cohort study (500 subjects) under varying degrees of measurement error

We can expect the screening of correlation of potential confounder and exposure by means of testing correlation between them to erode power: observed correlation in a sample can be very weak and imprecisely estimated even when there is true correlation in the population. This can be expected to most serious in small sample sizes and for weak true correlations. We examined this issue by examining loss of power due to the screening procedure in the case of *n* = 500 (small sample size in our simulation). We noted that our screening procedure excluded variables with little correlation (*ρ*≤ 0.1 for σ_x_^2^ = σ_z_^2^ = 0.25, and ρ≤ 0.2 for σ_x_^2^ = σ_z_^2^ = 1) with the exposure, and for *ρ* >0.3 these variables were nearly never being excluded (Fig. 8). When confounding was weak, the loss of power was more apparent. Thus, there appeared to be observable loss of power only for the weakest strength of confounding and when error variances are large.

### Application to the mercury, fish consumption, and depression example

As the degree of measurement error in the confounder increases, there is a precipitous drop in the proportion of analyses that would correctly suggest a null association (i.e. *p* > 0.05) of total blood mercury with depression (Table 1). If the noise-to-signal ratio (error variance in confounder (σ^2^)/variance of confounder) is at or below about half, roughly 80 % of the simulated analyses adjusting for fish consumption would correctly result in a null association of mercury and depression. We also observed that for the most part, if the confounder is forcibly adjusted for (as can be expected when a DAG confounder identification strategy is used) even while measured imperfectly, the effect estimates are noticeably much less confounded (i.e., RR closer to 1) as compared to the unadjusted RR of 0.83. Only when the noise-to-signal ratio is 1 or larger does adjusting for the miss-measured confounder make little to no difference. In other words only an extremely poorly measured confounder is not useful to adjust for in this setting.

**Table 1:** The proportion of 10,000 simulated adjusted analyses where a hypothesized null exposure-outcome association (RR_*ED*|*W*_) is indicated, after adjustment for a confounder *W* that is measured with different degrees of measurement error (Simulated change-in-estimate cutoff for Type I error <0.05 = 0.06 %)

Noise-to-signal ratio^a^	Proportion of results where RR_*ED*|*W*_ *p* >0.05 (%)	RR_*ED*_|_*W*_ (adjusted for confounder *W*)	
		Average	95 % Confidence Interval
0.10	100	0.99	0.97–1.01
0.25	100	0.98	0.96–1.00
0.50	91	0.96	0.94–0.98
0.55^b^	78	0.95	0.94–0.97
0.60	56	0.95	0.93–0.97
1.0	0	0.91	0.90–0.92
10^c^	0	0.83	0.83–0.83

^a^ratio of variance of measurement error relative to variance of confounder

^b^in practice, it is not possible to have such precise knowledge about the extent of measurement error, so any calculation of this sort is necessarily approximate and is meant as a guideline for selection of suitable method to measure a confounder, but we can say that error variance should be closer to 0.5 than to 0.6

^c^empirically determined to correspond to error in confounder hypothesized to exist in the data (*F*_*obs*_) and resulting in failure to control cofounding effect of RR_*ED*|*Fobs*_ = 0.83 for exposure to mercury

If we do not have sufficient knowledge to guide a theory-based confounder selection strategy, application of model selection cutoffs may be useful. In this specific setting, a simulation-derived CIE cutoff was small (0.06 %). If such a strategy is adopted, for the observed RR = 0.83 a change of 0.1 % after adjustment for *W* identifies it as a confounder, even though one can question whether such a small change is discernible from background noise in realistic applications. The degree to which it is important to remove such a degree of confounding depends on the specific application and can range from immaterial to important depending on the association of interest and the role it plays in whatever action is taken on the basis of effect estimation. However, it is clear that the CIE of 10 % is too coarse to detect confounding in this setting with the desired certainty.

**Fig. 9:**
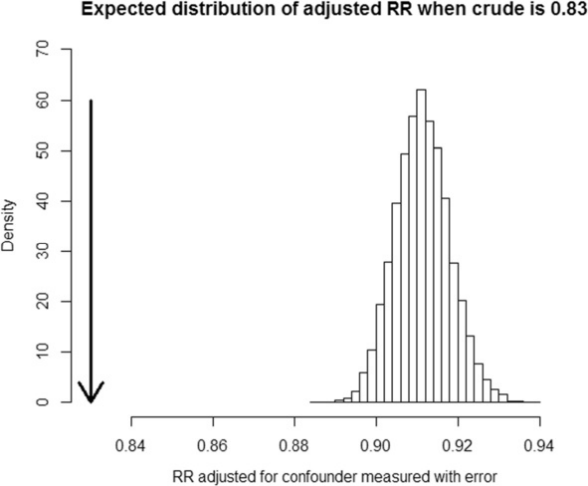
Anticipated estimates of the exposure-outcome association in the motivating example (see text for details) after adjustment for a miss-measured confounder when there is not a true exposure-outcome association. The unadjusted RR _ED|Fobs_ is 0.83 (i.e. confounded; indicated by the arrow), noise-to-signal ratio is 1 and all effects of confounder are expected to be not statistically significant (*p* >0.05); 10,000 simulations

Figure 9 shows the empirical distribution of the adjusted RR for the noise-to-signal ratio of 1 (median 0.909, inter-quartile range 0.905–0.914). We can see that we expect all estimates to be closer to null than the naïve and can therefore take the observed effects of that size to support the hypothesis that an apparent mercury-depression association is due to confounding by fish intake. Thus, even if residual confounding is not eliminated after adjusting for a mis-measured confounder, we can still determine whether evidence supports its role as a confounder. This clearly argues for a much more liberal rule for evaluating evidence for confounding, based on statistical grounds alone in the given motivating example, in the presence of measurement error in confounder, than is permitted by the 10 % CIE criteria. It also illustrates the peril of reliance on hypothesis testing: we do not expect to find a statistically significant effect of fish intake in the example illustrated in Fig. 9 and yet all “imperfectly” adjusted point estimates of RR are expected to be less biased than the crude value. This further argues for forcing a variable into a model if there is a theoretical reason to do so, regardless of whether a frequentist hypothesis test indicated an association.

## Discussion

### Overview of findings

Our study provides a framework that evaluates the performance of various empirical criteria with considerations of strength of causal and confounding effects, sample size, measurement errors in exposure and confounder, and types of outcomes. This framework is useful for study design and planning. During the stage of study design, researchers often need to choose, among many options, the tools for measuring the exposures and confounders. For example, they can choose among self-report or objectively-measured BMI, physical activity level, and dietary intake. Using the existing results from validation studies [10–13], researchers can make use of our framework to choose the most appropriate measurement tools that maintain a balance between the error of causal effect estimation and the total cost induced.

We were also successful in proposing a solution to the problem of false positive identification of risk factor as confounder in a finite sample. A simple evaluation of correlation between exposure and potential covariate achieved nearly perfect power. We emphasize that this is finite-sample problem that arises due to chance correlation, induced by the fact that *X* and *Z* are both related to *Y*, inducing a chance *X*-*Z* association via *Y* that had a tangible impact (i.e. on the order of simulated CIE cutoff) on estimate of effect of *X* on *Y* upon inclusion of *Z* in a regression model. This phenomenon disappeared when sample size was boosted and worsened in finite samples with large values of *β*_*Z*_ (details available on request). Even if *Z* is not identified as structural confounder, there is a good reason to include it in the final estimate to remove as much as possible confounding from the estimate of effect of *X*, however, in doing so, the understanding of problem under study is increased by gaining evidence for chance versus structural confounding by *Z*.

In practice, epidemiologists that use DAG to identify confounders try to distinguish between several plausible DAGs: this lies at the heart of confounder identification problem. However, all assumptions about plausible DAGs made by investigators can be wrong such that any choice among alternative causal structures does not reflect the true state of nature. Our work does not address such situation but it would be important to consider such a possibility in any truly improved approach to selection of confounder identification method; we believe that this is possible via simulations in a specific setting and allude to this in presentation of empirical example elaborated in this paper.

Given the complexity of factors that influence selection of confounder identification approach and tools even in our relatively simple settings, a practical approach is to customize simulations to reflect uncertainties about causal structures and imperfections of data when making such choices instead of reply on any *a priori* advice. Previous simulation studies showed that *a priori* advices such as significance criteria and 10 % CIE may lead to wrong decision of confounder adjustment when the exposure variable is error prone [21]. While a simulation-derived CIE criterion would change for every data situation, our study indicates that using simulations to inform model selection is both feasible and desirable during the study-planning stage, using information that most investigators possess: the knowledge of quality of instruments measuring exposure and confounders, as well as plausible strengths of the associations. It must be emphasized that we propose a solution to a problem that is sensitive to each specific application and, as such, our method is guaranteed to out-perform any general advice such as 10 % CIE, unless, by chance, simulation-based CIE are identical to 10 %, in which case our method will have identical performance relative to the general advice.

### Interpretations of findings from analysis of simulated studies

Despite complexities of patters of our results, they seem to exhibit several general tendencies. As the strength of confounding increases, the chance of identifying confounder, when present, also grows across constellations of measurement error, type of outcome and sample size, implying that all confounder identification strategies tend to perform better in picking up stronger effects. Most of our findings were consistent across different outcome types.

With respect to precision of the exposure-outcome association as measured by RMSE, there appear to two competing phenomena. As measurement error of the exposure increases, under the postulated classical measurement error model, for all regression coefficients to be attenuated towards null [7], such that whether an estimate is corrected for confounder or not would make little difference to its attenuated value that tends towards the null and a poorly-measured confounder would do little to remove true confounding in regression model. This would have the net effect of the RMSE to become independent of the strength of confounding or confounder-identification strategy. This is also indeed the case of real data example with confounder (fish consumption) very poorly measured. On the other hand, when measurement error is moderate (i.e. not so strong as to push estimate of the effect of exposure nearly to the null and to make any adjustment inconsequential to its magnitude), RMSE tends to decrease with superior confounder-identification strategy, i.e. correctly specifying the outcome model has tangible benefits. Unsurprisingly, RMSE is smaller for weaker confounding where there are fewer penalties for failing to adjust for confounder. These influence of RMSE create a rotation in RMSE versus the strength of confounding curves such that it may appear that RMSE declines with the strength of confounding with the rise of measurement errors, whereas in fact all we witness is the tendency for RMSE to become independent of measurement begin to dominate the tendency for RMSE to be larger when confounding is stronger.

The RMSE has to be interpreted with cautions, as it is a combination of squared bias and standard error of the causal effect estimate. In logistic regression (*n* = 2,000) with the largest examined measurement errors, 10 % CIE criteria leads to the smallest RMSE for strong confounding but it is essential to note that this is based almost exclusively on unadjusted estimates because confounder was almost never included in the final model (Fig. 3). We hypothesized that this phenomenon is due to the fact that with a poorly-measured exposure we expect the estimated causal effect would be zero (i.e., an RR of 1). The low RMSE in this case is the result of tight clustering of unadjusted attenuated estimates around wrong value of the effect estimate, a phenomenon that was previously described in theoretical work [14] that leads to over-confidence in wrong estimates in presence of measurement error. Another tendency that is acting on the observed RMSE is due to the fact that when exposure-confounder correlation increases, the standard errors of the maximum likelihood estimate of adjusted causal effect also increases, leading to the increase of RMSE.

We present two types of simulated CIE criteria: designed to control either type I or II errors. When the cutoff values of the two criteria are similar, we are in the fortunate situation where both types of error are controlled to the desired degree. In the synthetic examples that we evaluated, there appears to be little difference in RMSE for the two simulation-based CIE approaches. The two approaches diverged in success in confounder identification *per se,* but both outperformed significance testing and *fixed* cutoff of 10 % CIE.

### Depression-mercury-fish consumption example

The real data analysis of the NHANES data illustrated how researchers can make use of our framework to choose the most appropriate measurement tools that maintain a balance between the error of causal effect estimation and the total cost induced. By applying our framework, information about the degree of accuracy, validity, or measurement error one needs to achieve in order to obtain a less biased estimate of the causal relationship. For instance, our simulation result informs us that a noise-to-signal ratio of 0.5 or smaller for the variable fish consumption is desirable when we wish to estimate the causal relationship between total blood mercury and depression. In planning such an analysis, in the planning stage researchers should avoid using measurement tools that are only weakly correlated with the true nutrient intake, for example food-frequency questionnaire [10].

The real data analysis is founded on the assumption that here is no reason for exposure to mercury to be protective of risk of depression and therefore cofounding by fish consumption, known to be protective, is suspected. Of course one can assume that there is a small positive effect of mercury that is reversed by stronger negative confounding by fish consumption. One can repeat our simulations in such a way that mercury would have a causal risk factor, e.g. by weakening correlation of mercury with fish consumption or assuming weaker beneficial effect of fish consumption on the outcome. We did not explore this possibility in order to limit future considerations of plausible continuation of work in this setting as discussed in [18].

### Limitations

A major limitation of our study is that we considered a limited number of scenarios. If the assumptions of our simulations were violated, for example if the model is mis-specified, or if the errors do not follow normal distribution, the conclusions will be altered. Ideally, readers interested in implementing our approach should consider the validity of these assumptions and implement necessary modification to our R code. Another limitation is that the associations between exposure, confounder, and outcome maybe unknown. We believe it is possible (and desirable) to conduct analyses while acknowledging partial knowledge about causal structures, measurement error and exposure misclassification (e.g. using Bayesian framework [14, 22]): this may prove to be a promising extension to our work.

Another limitation is that we did not evaluate our framework under multiple confounders but this can be done in practice by slight modification of our R code. On the other hand, conceptually, one confounder may stand for multiple confounders that do not cancel each other out (e.g. see [8]), so our approach with one confounder should retain some generalizability.

One can argue that when measurement error is present, suitable analytical approach, for example regression calibration [23, 24] or simulation and extrapolation (SIMEX [25]), should be applied to remove its influence from inference before engaging in the discussion of suitable confounder identification strategy. This is certainly a sensible approach and the one that, to the extent possible, should be advocated. However, we wish to point out that measurement error correction is not routinely practiced by epidemiologists [26] and until such time that this changes, it is still relevant to consider how the historically and currently acceptable analytical strategies for model-building perform in practice.

## Conclusions

The impact of measurement error in a putative confounder on the selection of a correct disease model and testing of presence of confounding can be complex and difficult to predict in general. However, targeted investigations into how well one has to measure the confounder and how to interpret data contaminated by residual confounding are possible. They can inform and motivate work on better efforts to quantify risk factors and can help gauge the added value of such work. If an investigator plans to use regression methods to control for confounding and to empirically select among all plausible confounders the subset that can be evaluated with the data they are advised to determine what CIE criteria are most suitable in their situation [5]. While use of causal diagrams is certainly helpful in guarding against most egregious mistakes in model specification, causal diagrams may be incorrect or the putative confounder may have so much measurement error as to be entirely useless for adjustment purposes. Likewise, statistical considerations alone do not guaranteed selection of correct model. Therefore, one has to triangulate confounding using all available knowledge and tools [15].

We conclude by emphasizing that no *a prior* criterion developed for a specific application is guaranteed to be suitable for confounder identification in general. The customization of model-building strategies and study designs through simulations that consider the likely imperfections in the data would constitute an important improvement on some of the currently prevailing practices in confounder identification and evaluation.

### Ethics approval and consent to participate

NHANES 2009–2010 was approved by The National Center for Health Statistics Research Ethics Review Board; informed consent was obtained from all participants at the time of data collection and further consent for specific analyses of this publically available data is not needed.

### Availability of data and materials

The NHANES 2009–2010 dataset can be downloaded at http://wwwn.cdc.gov/Nchs/Nhanes/Search/nhanes09_10.aspx.

## Additional files

Additional file 1: R code for simulation study. (ZIP 8 kb) Additional file 2: R code for analysis of real data. (TXT 5 kb)
